# Exploring factors related to physical activity in cervical dystonia

**DOI:** 10.1186/s12883-015-0499-6

**Published:** 2015-12-01

**Authors:** Lena Zetterberg, Charlotte Urell, Elisabeth Anens

**Affiliations:** Department of Neuroscience, Section of Physiotherapy, Uppsala University, BMC, Box 593, 751 24 Uppsala, Sweden

## Abstract

**Background:**

People with disabilities have reported worse health status than people without disabilities and receiving fewer preventive health services such as counseling around exercise habits. This is noteworthy considering the negative consequences associated with physical inactivity. No research has been conducted on physical activity in cervical dystonia (CD), despite its possible major impact on self-perceived health and disability. Considering the favorable consequences associated with physical activity it is important to know how to promote physical activity behavior in CD. Knowledge of variables important for such behavior in CD is therefore crucial. The aim of this study was to explore factors related to physical activity in individuals with cervical dystonia.

**Methods:**

Subjects included in this cross-sectional study were individuals diagnosed with CD and enrolled at neurology clinics (n = 369). Data was collected using one surface mailed self-reported questionnaire. Physical activity was the primary outcome variable, measured with the Physical Activity Disability Survey. Secondary outcome variables were: impact of dystonia measured with the Cervical Dystonia Impact Scale; fatigue measured with the Fatigue Severity Scale; confidence when carrying out physical activity measured with the Exercise Self-Efficacy Scale; confidence in performing daily activities without falling measured with the Falls Efficacy Scale; enjoyment of activity measured with Enjoyment of Physical Activity Scale, and social influences on physical activity measured with Social Influences on Physical Activity in addition to demographic characteristics such as age, education level and employment status.

**Results:**

The questionnaire was completed by 173 individuals (47 % response rate). The multivariate association between related variables and physical activity showed that employment, self-efficacy for physical activity, education level and consequences for daily activities explained 51 % of the variance in physical activity (Adj R 0.51, F (5, 162) = 35.611, *p* = 0.000). Employment and self-efficacy for physical activity contributed most strongly to the association with physical activity.

**Conclusions:**

Considering the favorable consequences associated with physical activity it could be important to support the individuals with CD to remain in work and self-efficacy to physical activity as employment and self-efficacy had significant influence on physical activity level. Future research is needed to evaluate causal effects of physical activity on consequences related to CD .

## Background

It is common knowledge that regular levels of physical activity are important for health-related quality of life for all individuals [1]. People with disabilities have reported worse health status than people without disabilities and receiving fewer preventive health services such as counseling around exercise habits. This is noteworthy considering the negative consequences associated with physical inactivity and a sedentary lifestyle [2]. Physical activity can be defined as all bodily movement that derives from the contraction of the skeletal muscles and results in increased energy expenditure, for example while walking, cycling or participating in sports [3]. In contrast to the large number of publications on physical activity in movement disorders such as Parkinson’s disease, almost no research has been conducted on physical activity in cervical dystonia (CD), despite its possible major impact on self-perceived health and disability for the individual patient [4, 5]. Cervical dystonia is a neurological movement disorder with an estimated overall prevalence of 4.98 per 100 000. The disorder is characterized by excessive involuntary activity of the neck and shoulder muscles, leading to abnormal movements of the head and possibly pain [6, 7]. Although CD is generally non-progressive and limited to involuntary muscle spasms of the neck muscles the literature reveals multifaceted possible health consequences of CD. In addition to painful head and neck symptoms, disturbed sleep, limited walking, limited upper limb activity and psychosocial inconvenience have a negative impact on the patients’ daily and social life, as well as employment status [8, 9]. Fatigue has been reported to aggravate the symptoms of CD and to be one contributor to the variations in disability associated with CD [10]. Botulinum toxin A is the first-choice treatment to ameliorate pain and the disabling head postures [11]. Patients experience reduction in motor impairment following botulinum toxin. However, lack of reduction in disability despite satisfaction with botulinum toxin treatment indicates the need of complementary treatment [12]. Many patients request physiotherapy. The evidence of potential benefits of physiotherapy over the short term is growing [13]. However, the therapy described is mainly focused on restoring the head position and reducing disease-related complications and not on encouraging physical activity as part of a health promoting behavior [13, 14].

Multiple factors are linked to physical activity participation. The two most consistent correlates of physical activity in adults in the general population are health status and self-efficacy [15]. Self-efficacy is defined as the confidence that one can successfully execute the behavior required to produce an outcome, such as physical activity, and is a central concept in Social Cognitive Theory [16, 17]. Another type of self-efficacy is the degree of efficacy (i.e. self-confidence) to avoid a fall, known as fall-related self-efficacy [18]. Vacherot et al. [19] concluded that CD does not affect postural control. However, in the clinical physiotherapy setting individuals with CD often report that fear of falling hinders them from performing physical activity, indicating this is a contributory factor to reduced participation in physical activities. Additionally, age, gender, and social support are correlates of activity that have been reported in the general population [15]. It has also been suggested that a feeling of enjoyment is a key construct for explaining the motivation of sport and exercise participants [20]. To our knowledge the literature on physical activity in CD is almost non-existent. The only study available indicated that the level of physical activity was associated to quality of life and health [5].

Ellis et al. [14] propose a paradigm shift in the physiotherapy treatment for patients with disabilities caused by neurologic disorders. The shift means away from a tertiary prevention approach in which the emphasis is on restoring function and reducing disease-related complications, toward a secondary preventive approach with an integration of physical activity habits in health promotion which emphasizes sustained exercise and physical activity over the course of the disease. This secondary prevention approach includes the application of evidence-based behavioral change interventions. Thus, to acquire knowledge about variables important for a physical active behavior in CD is therefore crucial.

In this pre-study it was hypothesized that demographic characteristics such as age, gender, employment status, educational level and self-perceived aspects such as symptoms and consequences of CD, fatigue, self-efficacy to physical activity, falls self-efficacy, fear of falling, enjoyment and social support for physical activity would contribute to explain reasons for physical activity in CD. The aim of the study was therefore to explore the multivariate association between a model of demographic characteristics and self-perceived aspects and physical activity in CD.

## Methods

The study was performed as a cross-sectional survey with a descriptive and correlating design. One questionnaire was sent out by surface mail to all patients between 18-80 years diagnosed with CD and enrolled at neurology clinics in the region of Uppsala University Hospital, Sweden within a two weeks period in May 2011, (n = 369). Patients were excluded if they had dementia (n = 1), could not write or speak (n = 1) or were not diagnosed with CD (n = 2). All participants in the study provided written informed consent to participate. The study was approved by the Regional Ethical Review Board, Uppsala, Sweden, D-no 2010/278.

Physical activity was the primary outcome variable measured with the *Physical Activity Disability Survey – Revised* (PADS-R) [21]. This scale was used to measure self-perceived level of physical activity [22]. The scale includes six subscales; exercise, leisure time, physical activity, general activity, therapy, employment and wheelchair use for individuals with chronic illness and/or disability. For each scale the amount of physical activity during the previous week is reported. The ratings from the subscales are summed to give a total score. A higher score indicates a higher level of physical activity. No maximum score is set. For this study we divided the PADS-R scores of the group into > mean and < mean. Test-retest reliability has shown to be good [21].

The following measurements were the secondary outcome variables included in the model of self-perceived variables related to physical activity in CD.

### Cervical dystonia impact scale (CDIP-58)

This scale measures the self-perceived consequences of CD using eight health categories: “head and neck symptoms”, “pain and discomfort”, “sleep”, “upper limb activities”, “walking”, “annoyance”, “mood” and “psychological functioning” [8]. These eight subscales correspond to three conceptual domains, namely “symptoms”, “daily activities” and “psychosocial squelae”. High scores indicate a high impact of CD on the individual’s health. The maximum transformed score is 100. Rasch item analyses have been performed to test the validity of the scale [23]. CDIP-58 was more sensitive than comparable scales in detecting statistical and clinical changes in patients treated with botulinum toxin [24].

### Fatigue severity scale (FSS)

This scale measures the perceived level of energy and severity of fatigue. A total FSS is calculated by taking the mean of all statement scores and the maximum score is 7. A higher score indicates a more severe fatigue [25]. A FSS score ≥4 can be used as a cut-off score for the presence of fatigue [26]. The English and the Swedish versions have both been found to be reliable and valid [25, 27].

### Swedish version of the Exercise Self-Efficacy Scale (S-ESES)

The questionnaire includes items concerning confidence when carrying out regular physical activities and exercises. The scores are summed to give a total score of maximum 40, which indicates high self-efficacy to carry out physical activity and exercises. Satisfactory content validity as well as high internal consistency and scale integrity have been shown [28].

### Falls efficacy scale (Swedish version) (FES(S))

This scale measures the degree of confidence in ability to perform common daily activities without falling [29]. The scale is divided into two subscales 1) Personal Activities of Daily Living (PADL) and 2) Instrumental Activities of Daily Living (IADL). The items can be summed to a total score of maximum 130. A high score indicates higher fall-related self-efficacy. A score of less than 80 indicates reduced self-efficacy to perform daily activities without falling. The scale has shown high test-retest reliability [30].

A question of fear of falling on a nominal level (yes or no) was included in the questionnaire, namely “Are you afraid of falling?”.

*Enjoyment of physical activity* was measured using a scale developed by our research group. The scale used three statements for the experience of enjoyment during or shortly following physical activity of at least 10 min duration (e.g. walking): “I experience that it is fun to be physically active”, “I experience a feeling of wellbeing when I am physically active” and “I feel happy with myself when I am physically active”. The answers were graded on a visual scale (from 0 to 5), and were added to a total score ranging from 0 to 15, with 15 being the highest level of enjoyment.

*Social Influences on Physical activity (SIPA)* was used for measuring social support for physical activity [31]. The scale includes positive social influences (SIPApos) and negative social influences (SIPAneg) on physical activity. SIPApos includes questions on dimensions of companionship, informational and esteem support. SIPAneg includes questions on inhibitive, justifying and criticizing behaviours. To measure the occurrence of social influences of three different sources; family, friends and experts a 5-point scale is used. Content validity and reliability of the scale has been shown [31, 32].

*Demographic characteristics* were collected regarding participants’ age, gender, level of education, marital and employment status, time since diagnosis, smoking and Body Mass Index (BMI).

Swedish versions were used for all instruments. PADS-R and SIPA were professional translated according to “Guidelines for the process of cross-cultural adaption of self-report measures” [33]. Psychometric properties were taken into account for all measurements except for the “Enjoyment of physical activity” scale which was developed by our research group.

Descriptive statistics of demographic characteristics were summarized. Missing values for occasional data in scales with ordinal data were imputed with the median for the appropriate subscale when the level of missing data was <33 % of the subscale. If the amount of missing data was ≥33 % no summarizing was done [34]. For the different measures this was the case in 2.9 % at most.

Distribution of data was evaluated and differences between men and women were analyzed using Chi-square tests, Mann-Whitney *U*-test or Student’s *t*-test depending on the type of scale and normal distribution. Multivariate association analyses were conducted to investigate variables that might influence physical activity (dependent variable) for the whole sample, and females and males separately.

Finally, the five variables with highest *β* in the initial multivariate analysis were defined as the most important related independent variables. These were analyzed in an additional regression analysis in order to increase the strength of the models.

Analyses were utilizing SPSS (Statistical Package for Social Sciences, version 20). Statistical significance was set at *p* ≤ 05.

## Results

In total 173 individuals with CD (72.3 % female, mean age 61 years) with a median time since diagnosis of 14 years, were enrolled in the study giving a response rate of  47 %. The analysis of the non-responders (n = 181) showed a larger group of women than men, 59.2 % and 40.8 % respectively (p = 0.010). The men in the non-responders group were younger than the responders (55 ± 12 years) compared to 60 ± 10 years) (*p* = 0.010).

Demographic characteristics and secondary outcome measures are presented in Tables 1 and 2. No differences between the genders were found for most of the background variables except that more women were smokers, 26.4 % (*p* = 0.037). The female group also reported a higher level of fear of falling (*p* = 0.015), lower fall-related self-efficacy (*p* = 0.043) and higher pain and discomfort (*p* = 0.031). The mean physical activity level was 0.63 ± 1.23. Physical activity levels were lower in the female group; 68 % of the women versus 23 % of the men had an activity level less than the mean value.

**Table 1:** Sample characteristics with mean (SD), median (IQR), number or percent of total sample, %

Demographic characteristics	Total (n = 173)	Male (n = 48)	Female (n = 125)
Age (years)	61.1 (9.8)	61.5 (9.7)	60.2 (9.9)
Marital status			
Living alone	56 (32.7 %)	13 (27.7 %)	43 (34.7 %)
Living with partner	115 (67.3 %)	34 (72.3 %)	81 (65.3 %)
Living with children			
With children	18 (10.4 %)	9 (18.8 %)	9 (7.2 %)
Without children*	155 (89.6 %)	39 (81.2 %)	116 (92.8 %)
Education status			
Compulsory education	45 (26.3 %)	13 (27.7 %)	32 (25.8 %)
Upper-secondary education	79 (46.2 %)	23 (48.9 %)	56 (45.2 %)
Tertiary education	47 (27.5 %)	11 (23.4 %)	36 (29.0 %)
Employment status			
Unemployed	5 (2.9 %)	2 (4.2 %)	3 (2.4 %)
Employed fulltime	49 (28.7 %)	14 (29.2 %)	35 (28 %)
Retired	67 (38.7 %)	15 (31.2 %)	52 (41.6 %)
Student	11 (6.4 %)	1 (2.1 %)	10 (8 %)
Sickness benefit	37 (21.6 %)	14 (29.8 %)	23 (18.5 %)
Other	1 (0.58 %)	1 (2.1 %)	0
Time since diagnosis (years)	14 (7-21)	15 (9-21)	14 (6-20)
Smoker (yes)*	38 (22.4 %)	5 (10.9 %)	33 (26.6 %)
Body Mass Index (kg/m 2)	25.1 (22.9-28.2)	25.4 (23.5-27.5)	25.1 (22.5-28.2)

*Significant statistical difference between men and women (*p* = 0.05)

**Table 2:** Sample characteristics of outcome measures with mean (SD), median (IQR) or number

Outcome measure	Total	Male	Female
	n = 173	n = 48	n = 125
PADS-R			
Total	0.63 (1.23)	0.69 (1.18)	0.60 (1.25)
> mean	82	25	57
< mean	91	23	68
Subscales of CDIP-58			
Head and neck (HN)	70 (53-80)	67 (48-80)	70 (57-80)
Pain and			
discomfort* (PD)	58 (40-76)	48 (34-72)	60 (48-76)
Sleep (S)	45 (25-65)	42 (21-70)	45 (25-65)
Upper limb			
activities (UL)	50 (29-67)	43 (25-71)	51 (33-67)
Walking (W)	47 (27-71)	48 (24-66)	46 (31-71)
Annoyance (A)	45 (28-60)	35 (25-60)	48 (29-60)
Mood (M)	34 (20-51)	29 (20-49)	34 (23-52)
Psychosocial			
Functioning (PF)	46 (28-66)	48 (26-68)	46 (28-66)
Domains of CDIP-58			
Symptoms (HN,PD,S)	49 (30-61)	55 (41-74)	61 (48-73)
Daily activities (UL,W)	50 (31-67)	45 (26-70)	52 (33-67)
Psychosocial			
sequelae (A,M,PF)	42 (28-60)	38 (24-61)	42 (30-59)
FES			
Total*	124 (104-130)	128 (107-130)	120 (101-130)
Fear of falling			
Yes*	44	6	38
No	125	41	84
FSS			
Total score	4.1 (2.5-5-5)	3.8 (2.2–4.8)	4.4 (2.6–5.7)
>4	93	21	72
<4	79	27	52
Enjoyment of physical activity	14 (10-15)	12 (9-15)	14 (11-15)
SIPA (from family)	0 (0.0-0.2)	0.0 (0.0-0.3)	0.0 (0.0-0.2)

*Significant statistical differences between men and women *(p* ≤ 0.05)

Note: *PADS-R* Physical Activity Disability Survey. No maximum score is set. The score is divided into < and > score. *CDIP-58* Cervical Dystonia Impact Profile (subscales and related domains), max score 100. *FES* Falls Efficacy Scale, max score 130. *FSS* Fatigue Severity Scale, max score 7. Enjoyment of physical activity, max score 15. *SIPA* Social Influences on Physical Activity. No maximum score is set

The multivariate association analysis for the five most important related variables for the whole group showed that employment, physical activity self-efficacy, education level and consequences for daily activities explained 51 % of the variance in physical activity (Adj R^2^ 0.51, F (5, 162) = 35.611, *p* = 0.000). Employment and physical activity self-efficacy were the strongest contributors to the association with physical activity for the whole group (Table 3). When analyzing the five most important related variables for physical activity in the female group, the multivariate association analysis showed that employment, physical activity self-efficacy and education level explained 52 % of the variance in physical activity (Adj R^2^ 0.52, F (5, 115) =27.797, *p* = 0.000) with employment and physical activity self-efficacy the strongest contributors in the association. For the male group the analysis indicated that employment and age explained 53 % of the association (Adj R^2^ 0.53, F (5, 41) =10.777, *p* 0.000). Employment was the most strongly associated variable (Table 4).

**Table 3:** Multivariate association analysis fo the total sample with all related independent variables for physical activity in cervical dystonia and with the five variables with highest β in the initial multivariate analyses

Independent variables	All independent variables (n = 149)		The five variables with highest β in the initial multivariate analyses (n = 168)	
	β	*p*	β	*p*
Age	-0.008	0.918		
Gender	-0.033	0.601		
Employment	-0.431	0.000*	-0.448	0.000*
Children at home	0.078	0.250		
Education	0.149	0.016*	0.136	0.016*
Symptoms	0.063	0.471		
Daily activities	-0.155	0.173	-0.146	0.032*
Psychosocial sequeale	-0.004	0.970		
Fatigue	0.003	0.968		
Physical activity self-efficacy	0.139	0.120	0.226	0.001*
Fall-related self-efficacy	0.036	0.665		
Fear of falling	0.057	0.480		
Social support (from family)	0.104	0.104		
Enjoyment of physical activity	0.107	0.124	0.096	0.113
	Adj R2 = 0.494, (F 14, 134) = 11.303, *p* = 0.000		Adj R2 = 0.509, F(5, 162) = 35.611, *p* = 0.000	

*p = 0.05

**Table 4:** Multivariate association analyses identifying the five variables with highest β in the initial multivariate analyses for physical activity in cervical dystonia for males and females, respectively

Independent variables	Male (n = 47)		Female (n = 121)	
	The five variables with highest β in the initial multivariate analyses		The five variables with highest β in the initial multivariate analyses	
	β	*p*	β	*p*
Age	-0.247	0.052*		
Employment	-0.473	0.001*	-0.401	0.000*
Children at home			0.102	0.113
Education	0.097	0.376	0.170	0.009*
Daily activities			-0.131	0.085
Fatigue	-0.225	0.076		
Physical activity self-efficacy			0.330	0.000*
Fall-related self-efficacy	0.084	0.474		
	Adj R2 = 0.528, F(5,115) = 27.797, *p* = 0.000		Adj R2 = 0.515, F(5, 41) = 10.777, *p* = 0.000	

*p = 0.05

## Discussion

This study provides a new contribution to the literature in understanding what variables affect physical activity levels in patients with CD. Of the various factors studied, employment emerged as the most strongly related variable for physical activity for the total group, as well as for the male and female groups, respectively. Physical activity self-efficacy emerged as the second most strongly related variable for the total group, and also for the female group. For the male group age emerged as the second most important variable to influence physical.

Prior findings indicate that employment status is frequently affected in CD [35]. Molho et al. [9] concluded that presence of pain was significantly associated with adverse employment outcomes and indicated a strong trend with regard to complete unemployment. Pain in CD is commonly considered a consequence or a symptom of the pathophysiological lesion, and is analyzed and treated accordingly [36–39]. Botulinum toxin treatment helps many CD patients to remain in employment [35].

Pain, measured with CDIP-58 [8] in this study, was a significantly worse symptom for the female group than for the male group. However, our findings indicate that employment status is associated with physical activity independent of the presence of pain. Being employed may contribute to physical activity on a daily basis, which may reduce pain and in so doing contribute to the possibilities of remaining in work. On the other hand, the individuals who were employed fulltime may have had less pain and less negative consequences related to CD, which this study could not identify. Questions about working conditions should be considered in future research to determine the value of working conditions in relation to physical activity levels. Our study did not include data on perceived pain relief following BTX treatment, which could be a contributing factor to the results [35]. The relationships between physical activity, pain and employment in CD need to be further investigated.

Self-efficacy is considered to be the strongest determinant of behavior, including physical activity, in the general population [40]. There is now accumulating evidence that this is also so among patients with Multiple Sclerosis [41]. This is in accordance with our results. Consequently, future research should consider developing and testing interventions that include self-efficacy as a modifiable factor to stimulate physical activity participation in CD. It is worth noting that age was not a limiting factor for physical activity for the women, but it was for the male group in this study. Older women have been shown to increase their interest in health-promoting behaviors, such as exercising [42], which could help to explain this result.

The impact of CD for the individual patient, was presented with scores from CDIP-58 [8]. Two out of the three conceptual domains of CDIP-58, representing the impact of CD on daily activities and psychosocial functioning, had higher scores, i.e. showed higher impact on health compared with scores presented earlier. The higher scores of CDIP-58 indicate that this survey rather included the individuals with higher disability due to CD than the opposite [5, 8]. However, one earlier study indicates that the severity of dystonic symptoms was not associated with an impact on either quality of life or health indicating that the severity of dystonic symptoms was not a crucial factor for quality of life, irrespective form of dystonia [5]. Other conditions such as ageing and/or medication could have deleterious effects on physical activity. Possible medications and co morbidity affecting physical activity in general and/or possible co morbidity as well as time since last botulinumoxin injection and time of completion of the survey were not asked for due to the problematic way of asking this in a self-reported questionnaire and the subsequent risk of biased analyses. Treatment with botulinumtoxin reduces dystonic symptoms however, if the effect of botulinumtoxin injections is a predictor of physical activity needs to be explored.

The response rate in this study was less the 50 %. The low response rate is problematic e.g. those who did not respond may be physically less active, psychologically more inert/passive or more depressed or on the other hand not. The low response rate also limits generalization of the results for the entire CD population. However, gender and mean age of the study group was consistent with other studies describing individuals with CD [43]. The questionnaire included eight measurements. Fewer questions may have increased the chance of obtaining a higher response rate. However, this survey could not have been condensed into just a few questions, as several known variables related to physical activity had to be included [15]. The different selection of study populations in these studies (patients enrolled at neurological clinics vs. members of a dystonia patient association) may contribute to explaining why our study group revealed a poorer health status. Patient organizations may attract a particular subsection of the overall patient population. We argue that the representativeness was guaranteed by selection patients enrolled at a university neurology clinic and associated regional hospitals.

The mean physical activity level for the whole group, measured with PADS-R [21], was 0.63 ± 1.23 which is more than three times higher than in a sample of patients with Multiple Sclerosis, where the mean PADS-R [21] score was 0.18 ± 1.475 [44]. The comparative results may not be unexpected as CD mainly affects the neck and shoulder muscles. However, it is not yet known which value for PADS-R [21] is equivalent to the recommendations for physical activity level issued by the World Health Organization, meaning that we do not know the level of physical activity for our study group in comparison with current recommendations. No maximum PADS-R score is defined [21]. In order to understand the level of physical activity in the study group we divided the PADS-R scores [21] into scores above and below the mean.

This was a cross-sectional study, with all variables measured using a self-reporting questionnaire. Most variables represented the respondents’ concepts and for that reason results were only accessible by self-reporting. Measures were chosen based on recognized reliability and validity in CD and in other chronic conditions, recommendations and clinical experience. However, the questions can be discussed. Physical activity can be objectively assessed by accelerometry [45], which should be of value for optimizing internal validity in future research. The measurement “Enjoyment of physical activity” was developed by our research group. The variable of enjoyment did not appear as a predictor for physical activity in our study group. However, the enjoyment scale consequently still lacks psychometric properties and the enjoyment results should be interpreted with caution.

## Conclusions

This study highlights the fact that being employed and having physical activity self-efficacy explains a large proportion of the variation in physical activity in CD. Considering the favorable consequences associated with physical activity for all individuals it is important that the individual patient with CD is supported to remain in employment and to be physically active. However, the implications of the result must be viewed with caution due to the low response rate. To support in self-efficacy, competence in behavioral medicine is required so that cognitive behavioral strategies and medical treatment for developing personalized goals for physical activity level can be combined. Future research needs to perform tailored interventions in randomized trials to evaluate casual effects of physical activity on consequences related to CD.

### Availability of supporting data

Contact the corresponding author if requested.

## Abbreviations

CD: Cervical dystonia
PADS-R: Physical Activity Disability Survey – Revised
CDIP-58: Cervical Dystonia Impact Scale
FSS: Fatigue Severity Scale
S-ESES: Swedish version of the Exercise Self-Efficacy Scale
SIPA: Social Influences on Physical activity
SIPAneg: Social influences on physical activity- negative social influences
SIPApos: Social influences on physical activity- positive influences
BMI: Body Mass Index
